# Dynamics and Flexibility of Human Aromatase Probed by FTIR and Time Resolved Fluorescence Spectroscopy

**DOI:** 10.1371/journal.pone.0082118

**Published:** 2013-12-11

**Authors:** Giovanna Di Nardo, Maximilian Breitner, Sheila J. Sadeghi, Silvia Castrignanò, Giampiero Mei, Almerinda Di Venere, Eleonora Nicolai, Paola Allegra, Gianfranco Gilardi

**Affiliations:** 1 Department of Life Sciences and Systems Biology, University of Torino, Torino, Italy; 2 Department of Experimental Medicine and Surgery, University of Rome ‘Tor Vergata’, Italy; National Institute for Medical Research, Medical Research Council, London, United Kingdom

## Abstract

Human aromatase (CYP19A1) is a steroidogenic cytochrome P450 converting androgens into estrogens. No ligand-free crystal structure of the enzyme is available to date. The crystal structure in complex with the substrate androstenedione and the steroidal inhibitor exemestane shows a very compact conformation of the enzyme, leaving unanswered questions on the conformational changes that must occur to allow access of the ligand to the active site. As H/D exchange kinetics followed by FTIR spectroscopy can provide information on the conformational changes in proteins where solvent accessibility is affected, here the amide I region was used to measure the exchange rates of the different elements of the secondary structure for aromatase in the ligand-free form and in the presence of the substrate androstenedione and the inhibitor anastrozole. Biphasic exponential functions were found to fit the H/D exchange data collected as a function of time. Two exchange rates were assigned to two populations of protons present in different flexible regions of the protein. The addition of the substrate androstenedione and the inhibitor anastrozole lowers the H/D exchange rates of the α-helices of the enzyme when compared to the ligand-free form. Furthermore, the presence of the inhibitor anastrozole lowers exchange rate constant (*k_1_*) for β-sheets from 0.22±0.06 min^−1^ for the inhibitor-bound enzyme to 0.12±0.02 min^−1^ for the free protein. Dynamics effects localised in helix F were studied by time resolved fluorescence. The data demonstrate that the fluorescence lifetime component associated to Trp224 emission undergoes a shift toward longer lifetimes (from ≈5.0 to ≈5.5 ns) when the substrate or the inhibitor are present, suggesting slower dynamics in the presence of ligands. Together the results are consistent with different degrees of flexibility of the access channel and therefore different conformations adopted by the enzyme in the free, substrate- and inhibitor-bound forms.

## Introduction

Cytochrome P450s are heme-containing monoxygenases interacting with a wide range of endogenous and exogenous substrates. They are involved in several processes including vitamin and steroid biosynthesis as well as detoxification of xenobiotics [1]. These enzymes recognise a wide range of molecules and possess differently shaped and sized active sites, in some cases accommodating even more than one molecule [2]. As an example, human cytochrome P450 3A4 was shown to bind simultaneously two molecules of the inhibitor ketoconazole in the active site of the protein at the same time [2]. The crystal structures of some mammalian cytochromes P450 in the presence and in the absence of substrates or inhibitors have revealed that these enzymes can adopt open and closed conformations [3], [4]. For the bacterial P450 BM3, it was shown that helix movements and conformational changes take place in the heme domain upon fatty acid binding [5]–[8]. For the heme domain of this protein, different crystal structures in the ligand-free form are present and revealed that there is a rearrangement of helices, suggesting the presence of different conformers in solution with a different degree of accessibility of the active site [9]. Very recently, it has also been shown a crucial role of the redox partner putidaredoxin in shifting the bacterial cytochrome P450cam toward the open conformation and favour proton-coupled electron transfer [10].

In the case of human aromatase, the cytochrome P450 acting in the steroidogenesis by converting androgens in estrogens [11]–[13], the crystal structure in complex with androstenedione has revealed a very compact active site where the steroid molecule snugly fits [14] and the catalytic pocket is optimized for androgens binding. Its size (around 400 Å^3^) is considerably smaller than that of other P450 enzymes involved in drug and xenobiotic metabolism such as 3A4 (530 Å^3^) or 2D6 (540 Å^3^) [15], [16]. Furthermore, the recently solved crystal structures of aromatase in complex with steroidal inhibitors revealed that some conformational movements at the entrance of the substrate access channel take place, but the overall structure is very similar to the one solved in the presence of the substrate androstenedione, with a root mean square deviation (RMSD) of 0.3 Å for the backbone atoms [17]. Aromatase has not yet been purified in a stable form and crystallized in absence of any ligand or in the presence of a non-steroidal inhibitor. Molecular dynamics simulations have shown that, although the catalytic cleft is very rigid, fluctuations of the F-G loop together with bending and twisting motions contribute to open and close the access channel of aromatase to allow the entrance of ligands [18], but these *in silico* findings are not at present supported by experimental data about the dynamics and flexibility of this important enzyme.

Since conformational changes often play a crucial role in substrate/inhibitor binding and catalysis, different approaches and techniques have been applied to study the dynamics and flexibility of cytochromes P450. They include NMR [19]–[21], crystal structures in complex with the so-called molecular wires (22), kinetics and termodynamic studies [23], [24], pressure-induced UV-vis spectral transitions [25], [26], H/D exchange coupled with mass spectrometry [27]–[29] and molecular dynamics simulations [30]. These studies have demonstrated that depending on the nature of the ligand and on the P450 studied, multiple conformers can simultaneously exist in solution upon ligand binding in some cases [19], [22], [31]–[32] whereas, in other cases, the ligand can lock the enzyme into a single conformational state and increase the rigidity of the protein [33].

In a previous work, we demonstrated by a combined EPR and Hyperfine Sublevel Correlation spectroscopic (HYSCORE) analysis that the inhibitor anastrozole binds to the heme iron of aromatase via the triazole moiety and must therefore be able to access the catalytic pocket of the enzyme [34]. In this work, we report a straightforward protocol developed to obtain a protein stable even in absence of any ligand and therefore suitable to be studied by ATR-FTIR and time resolved fluorescence to investigate how the dynamics and flexibility of the protein change when a substrate or a non-steroidal inhibitor is added in the catalytic cleft.

FTIR is a powerful tool to follow H/D exchange kinetics for a wide range of proteins [35]. The time-course of the isotope exchange can be followed by FTIR transmission experiments either by dissolving lyophilized protein in deuterated water, or by flowing deuterium-saturated nitrogen gas through a cell containing the protein deposited on the germanium crystal of an attenuated total reflection (ATR) device. Since mammalian cytochromes P450 are membrane proteins difficult to manipulate and lyophilized, the second option was applied for the first time to a human cytochrome P450 that is an important drug target for estrogen-dependent cancer.

## Materials and Methods

### Cloning, expression and purification of recombinant human aromatase (rArom)

Human aromatase cDNA was N- and C-terminally modified and cloned directly into a pCW Ori+ vector containing an IPTG-inducible Tac promoter and an ampicilllin-resistance gene. Amino acids 1–39 at the N-terminus of the protein sequence were replaced by a 10 residues fragment containing positively charged and hydrophilic amino acids (MAKKTSSKGR) as previously described by Hong and co-workers [36]. At the C-terminus a four-histidine tag was introduced to facilitate purification by affinity chromatography.

The expression of recombinant aromatase (rArom) was carried out in *Escherichia coli* strain DH5α. Competent cells were transformed with the rArom clone and used to inoculate a 5 ml Luria-Bertani (LB) liquid culture containing 100 µg/ml of ampicillin. After 16 hours growth at 37°C, 5 ml of the liquid culture were transferred to shaking flasks containing 0.5 L of terrific broth (TB) medium supplemented with 100 µg/ml ampicillin. Cells were grown to an OD_600_ of 0.5–0.8 before induction with 1 mM IPTG and incubated with 0.5 mM δ-aminolevulenic acid for 48 hours at 28C°. Cells were then harvested and resuspended in 100 mM potassium phosphate buffer pH 7.4 containing 20% glycerol, 1 mM β-mercaptoethanol, 0.1% Tween-20. After 60 minutes of stirring at 4°C, 1 mM of phenylmethylsulfonyl fluoride and 1 mg/ml lysozyme were added. The detergent (Tween 20) concentration was then raised to 1% and cells were disrupted by sonication on ice. After ultracentrifugation, the supernatant containing the soluble rArom was loaded onto a diethylaminoethyl ion-exchange column (DEAE-Sepharose Fast-Flow, GE healthcare) followed by a Nickel-ion affinity column (Chelating sepharose Fast-Flow, GE healthcare). A linear histidine gradient (1–40 mM) was applied to elute the protein. Aromatase containing fractions were pooled and histidine removed using a 30 kDa Ultracel centrifugal device (Amicon, Millipore). The protein was stored at −20°C.

### UV-vis spectroscopy

The P450 content of purified aromatase samples was measured using reduced CO-difference spectra [37]. The CO binding assay was performed in a 1 cm cuvette cell by following the increase of the signal at 450 nm after reduction of rArom with sodium dithionite and bubbling with CO for 10 minutes.

The absorbance at 450 nm was recorded by an Agilent 8453E UV-Vis spectrophotometer and the iron (II)-CO complex amount was estimated by using an extinction coefficient at 450 nm of 91,000 M^−1^ cm^−1^ [37].

Binding of androstenedione and anastrozole to the active site of rArom were monitored spectroscopically as a shift of the absorbance maximum from 418 nm to 394 nm for androstenedione and from 418 nm to 422 nm for anastrozole. Increasing concentrations of androstenedione or anastrozole (from 0.1 µM to 10 µM) were added to rArom in 100 mM potassium phosphate pH 7.4, 20% glycerol, 0.1% Tween 20, 1 mM β-mercaptoethanol. After equilibration the spectrum was recorded by a diode array UV-vis spectrophotometer (Agilent 8453E, Agilent Technologies). The dissociation constant K_D_ was calculated using the equation:
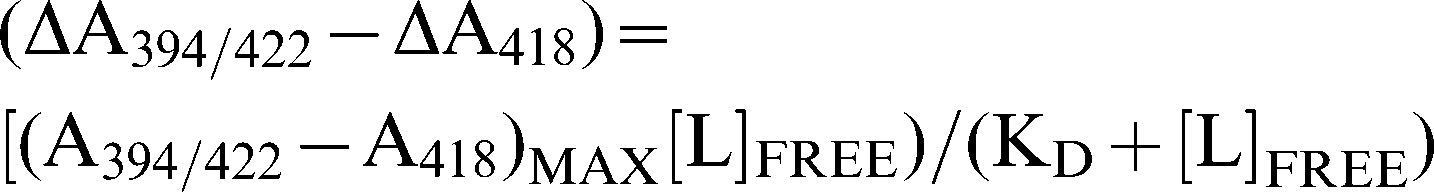
(1)where [L]_FREE_ is [L]added-[EL] and [EL] = (ΔA_394/422_−ΔA_418_)[P450]/(ΔA_394/422_−ΔA_418_)_ MAX._

The samples of rArom in complex with the substrate androstenedione and the inhibitor anastrozole were prepared through an overnight incubation of the protein with 10 µM of androstenedione or 1 µM of anastrozole at 4°C.

### Activity assay

Aromatase activity assay was carried out by the water release method [38]. The protein (30 nM) purified in absence of substrate was incubated with 30 nM of human cytochrome P450-reductase (Invitrogen) with 500 nM of the substrate 1-β-H^3^-androstenedione (Perkin Elmer). The reaction was then started by the addition of 1 mM NADPH and the mixture incubated for 10 minutes at 37°C. The reaction was then stopped by the addition of trichloroacetic acid (50% v/v) and the protein precipitated by centrifugation. The supernatant was then applied to a Strata X SPE column (Phoenomenex) for the separation of the androstenedione from the water phase.

The water phase was then counted by a Tri-Carb 2100TR liquid scintillation analyzer (Packard Bioscience). Control experiments were performed by omitting cytochrome P450-reductase to the reaction mixture for each substrate concentration.

### Thermal stability

Circular dichroism (CD) spectra of rArom were collected in the far-UV range (200–250 nm) at a concentration of 1 µM using a 0.1 cm pathlength cell. Spectra were recorded at room temperature on a Jasco-815 instrument (Jasco Instruments). Both the buffer and the substrate androstenedione resulted not to affect the spectra at the concentration used for the experiments with rArom plus substrate.

Thermal denaturation experiments were performed by raising the temperature from 25 to 70°C and allowing the protein sample (1–3 µM) to equilibrate for 3 minutes at each temperature before recording the spectrum. The CD signal at 208 nm was plotted as function of temperature and the data fitted to a single step transition curve to derive the melting temperature (T_m_). Five independent curves were considered for each sample (rArom, rArom+androstenedione and rArom+anastrozole) to derive the mean and the standard deviation for each experimental point.

### Fluorescence spectroscopy

Static fluorescence measurements were carried out using a ISS spectrofluorometer (ISS, Champaign, IL, USA). The protein sample (5 µM) was excited at 293 nm and the emission spectrum recorded from 310 and 450 nm.

Dynamic fluorescence measurements were carried out on 3–5 µM protein samples with a K2 ISS spectrofluorometer (ISS, Champaign, IL, USA) with a laser diode source. The excitation wavelength was 300 nm, and the emission signal was collected through a WG320 cut-off filter.

### ATR-FTIR spectroscopy

Infrared spectra of aromatase were acquired using attenuated total reflectance (ATR) tool (Harrick Scientific Products, Pleasantville, NY) in a Bruker Model Tensor 27 FT-IR spectrometer (Bruker Instruments, Billerica, MA) with a scan velocity of 10 kHz and a resolution of 4 cm^−1^. A thin protein film is deposited on an internal reflection element. All spectra were collected from 4000 to 800 cm^−1^. Data were collected in triplicate and spectra were averaged, using the Opus software (Bruker Instruments, Billerica, MA). Spectra of the protein were corrected by subtraction with control samples acquired under the same scanning and temperature conditions. The amide I band was corrected for the contribution of aromatic amino acids and then deconvoluted using PeakFit software (SPSS Inc., USA).

### Kinetics of H/D exchange

For hydrogen/deuterium exchange, nitrogen gas was saturated with ^2^H_2_O by bubbling through a bottle containing 200 ml of ^2^H_2_O.

The protein sample (20 µl of 100–150 µM) was placed on the germanium crystal of the ATR tube in a home-made cell. At the zero time, the tubing carrying nitrogen saturated with ^2^H_2_O was connected to the cell. Spectra were recorded at 24°C every 60 seconds for the first 10 minutes and at 8 minutes intervals up to 200 minutes. Twenty scans were recorded and averaged for each time point.

The H-D exchange was monitored by following the changes in the amide I band, In order to dissect the contribution of the different elements of the secondary structure, second derivative spectra were considered. The relative intensity change was considered and plotted as function of time. Fitting to exponential curves were performed by using Sigma Plot software. Statistical analysis for significance on the H/D exchange rate constants was performed by T-test.

## Results

### Preparation, stability and activity of ligand-free form of rArom

Although human aromatase has been cloned and expressed in heterologous systems by other groups [36], [39]–[42], many difficulties have been encountered in obtaining a stable ligand-free enzyme. For this reason the procedures reported to date involve the addition of substrates or inhibitors during the purification process with the aim of stabilising the active folded state of the protein. Though viable, this approach leads to a difficulty in the full removal of the ligands that results in sample heterogeneity due to the co-existence of different spectroscopic species. As a consequence, the first objective of the present work is to present data from a new system that includes expression in a pCW vector in *E.coli* DH5α and a new purification procedure able to give 15–20 mg of pure protein per litre of liquid culture in a stable ligand free-form of the enzyme. Very recently, the method has also allowed to obtain crystals from the protein co-purified with the substrate androstenedione and the crystal structure of recombinant aromatase was solved and demonstrated an almost complete superimposition with the placental one [43].

**Figure 1:**
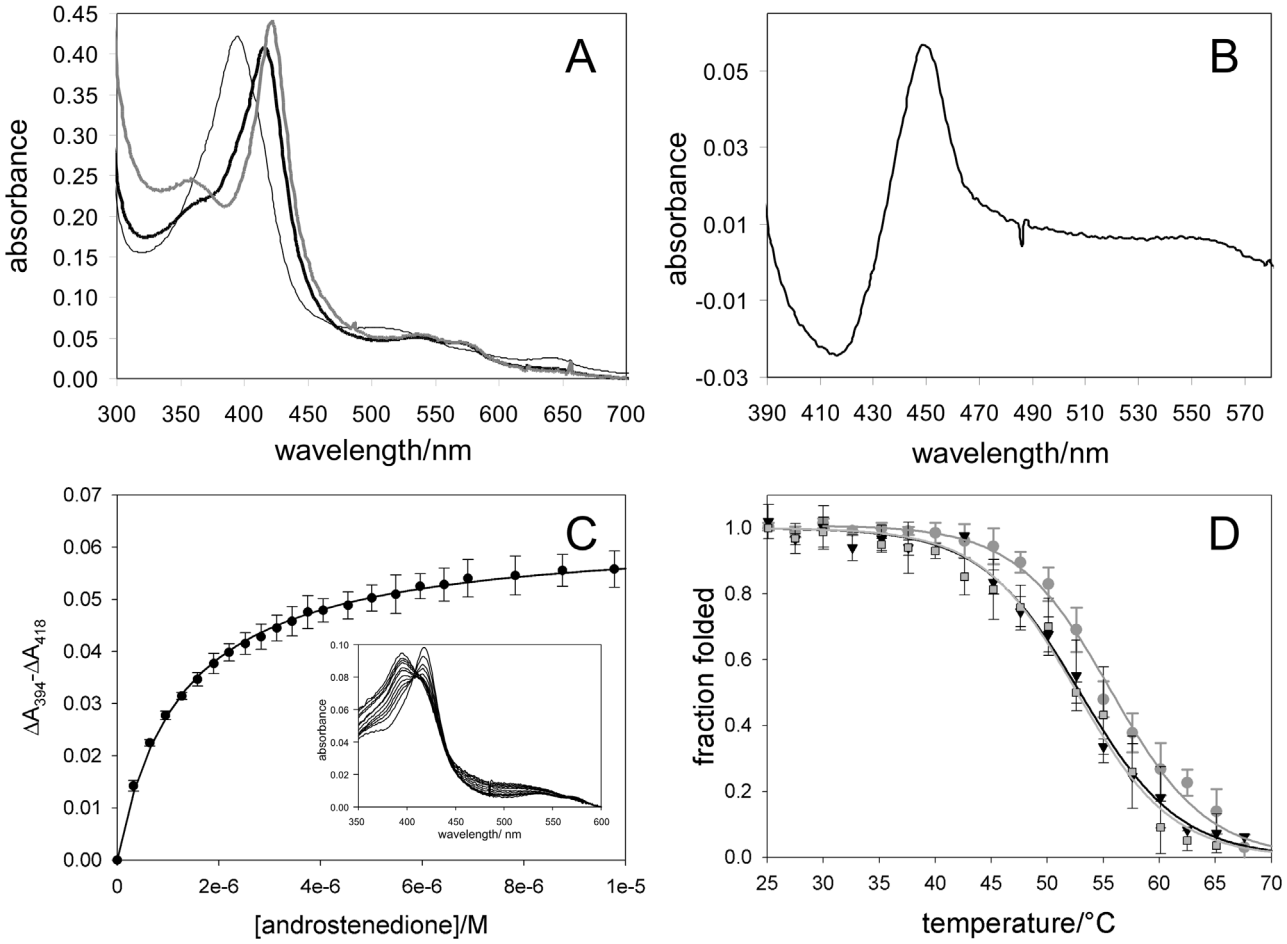
Absorption spectroscopy of rArom. A) Visible absorption spectrum of 3.8 µM rArom in the absence of substrate (thick black line) and in the presence of 10 µM of the substrate androstenedione (thin black line) and 1 µM of the inhibitor anastrozole (grey line). B) Difference spectrum of Fe(II)-CO minus Fe(II) of rArom purified in absence of any substrate/inhibitor. C) Binding curve of androstenedione (inset: spectra recorded during substrate titration). D) Thermal denaturation of rArom in the absence (squares), and in the presence of the substrate androstenedione (circles) or the inhibitor anastrozole (triangles) monitored as a decrease in ellipticity at 208 nm in the far-UV CD spectra. The data were fit to a two-state transition to obtain T_m_ for rArom in the absence (light grey curve), and presence of the substrate androstenedione (dark grey curve) or the inhibitor anastrozole (black curve).

The purified protein shows the typical Soret maximum at 418 nm and the α and β bands at 535 and 571 nm, respectively (Figure 1A).

The difference spectrum Fe(II)-CO minus Fe(II) of a sample of aromatase purified in the absence of any ligand shows the typical peak at 450 nm whereas no P420 species is detected (Figure 1B).

**Figure 2:**
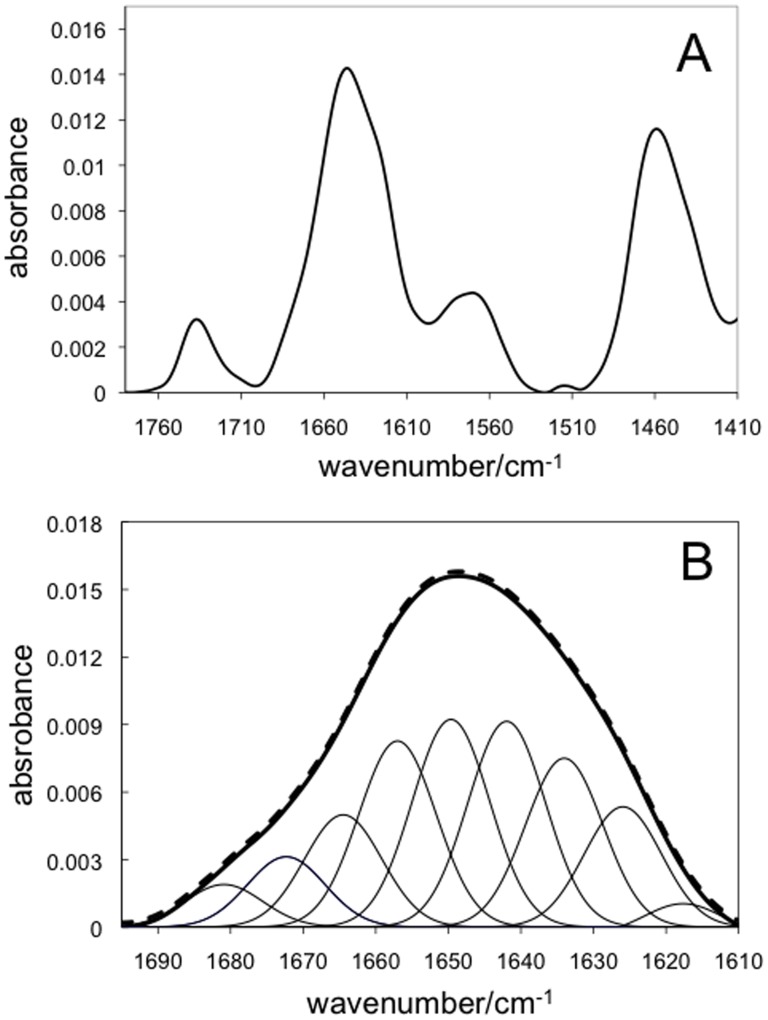
FTIR spectrum of rArom in D_2_0. A) FTIR spectrum of rArom obtained by depositing 20 µl of rArom (135 µM in 100 mM KPi pH 7.4 + 20% glycerol + 0.1% Tween 20 + 1 mM β-mercaptoethanol) on the germanium crystal of the ATR accessory. B) Deconvolution of the amide I band signal (thick line) into its nine components (thin lines). The experimental (solid thick line) and the predicted (dashed line) spectra are shown.

Thermal unfolding studies were performed and followed by far UV circular dichroism (CD) spectroscopy. The CD signal at 208 nm was recorded in the temperature range 25–70°C after allowing the sample to equilibrate for 3 minutes at each temperature (Figure S1). The unfolding process leads to a typical cooperative single step sigmoidal curve for all the samples analysed (Figure 2D). The unfolding process results irreversible suggesting protein aggregation during denaturation and problems related to the loss of the heme as previously observed for other cytochromes P450 [44] Therefore the unfolding transition temperature is presumed as apparent [45] and determined to be 52.9±0.3°C for the free rArom that indicates a stable protein on which to perform binding studies.

All together these results show that rArom was indeed purified in a stable form in the absence of added substrates or inhibitors during the purification procedure. This enzyme is therefore further characterised in terms of ability to bind a substrate and an inhibitor, stability in the presence of ligands and catalytic activity.

After incubation of the protein with saturating amounts of the substrate androstendione (10 µM) or the inhibitor anastrozole (1 µM), the typical spectral transitions of cytochromes P450 for type I (androstenedione) and type II (anastrozole) ligands are observed (Figure 1A).

Upon androstenedione binding the Soret maximum completely shifts to 394 nm and the α and β bands to 513 and 543 nm respectively, with a charge transfer band at 643 nm typical of the iron in a high spin state (Figure 1A). This shift reflects the low-to-high spin transition of iron due to the displacement of the water molecule acting as the six^th^ axial ligand [46]. The Soret maximum of aromatase in the presence of anastrozole is detected at 422 nm (Figure 1A), with α and β bands at 535 and 571 nm, respectively. Titrations with increasing amounts of the substrate androstenedione (Figure 1C) and the inhibitor anastrozole were performed to measure the dissociation constants that result 1.2±0.1 µM and 0.29±0.03, respectively.

The stability of the ligand-bound protein was studied by CD thermal unfolding studies performed in the same conditions of the ligand-free form described above.

The unfolding process led to typical cooperative single step sigmoidal curves from which unfolding transition temperatures (T_m_) were found to be 55.7±0.3°C and 52.6±0.4°C for the substrate– and inhibitor-bound forms respectively, indicating a small stabilizing role of the substrate. As these values are very close to those found for the free-enzyme, they further demonstrate that the protein was purified in a stable form even in the absence of a ligand.

The specific activity of rArom purified in absence of any substrate/inhibitor resulted 19.8 nmol/min/mg of protein. This value is comparable to the one of the protein purified in presence of androstenedione (16.4 nmol/min/mg of protein) and to that reported for placental wild type aromatase (10 – 100 nmol/min/mg of protein) (14). The presence of 1 µM of anastrozole resulted in an almost complete inhibition of the protein activity (<5%).

### ATR-FTIR spectroscopy and H/D exchange

The FTIR spectrum of rArom after 200 minutes of flowing ^2^H_2_O-saturated nitrogen gas in the cell containing the protein sample deposited on the ATR surface is shown in Figure 2A. The band at 1648 cm^−1^ is assigned to the amide I primarily due to stretching of the peptide C = O group, while the band at 1455 cm^−1^ is assigned to the amide II that corresponds to the N–H bending coupled with C–N stretching [35]. The signal at 1739 cm^−1^ was previously detected in P450_cam_ and was assigned to the C = O stretch vibration of the carboxyl group of aspartic and glutamic acid residues or to the heme propionates [47]. The signal arising from tyrosine residues is also detected at 1517 cm^−1^ [48].

The FTIR spectra of the samples of aromatase in complex with the substrate and the inhibitor do not show any significant difference compared to the ligand-free sample. However, possible modifications in the protein secondary structure induced by the presence of the substrate/inhibitor were investigated by analysing the amide I band, that it is used to estimate the amount of the secondary structure elements in proteins.

Fitting of the amide I signal to a sum of nine Gaussians was carried out as previously reported in the literature for other cytochromes P450 (Figure 2B) [49]. Comparison of the raw data (solid line) with the sum of the fitted Gaussians (dashed line) shows a very good agreement. The areas for each component lead to the assignments reported in table 1 that show that the data are within the range of those found in the literature confirming the viability of our protein samples. The results also show that addition of androstenedione or anastrozole does not alter the rArom secondary structure, as also confirmed by the far UV region of the CD spectra of free and bound rArom that show a complete overlap (data not shown).

**Table 1:** Amount of secondary structure elements (%) present in rArom and comparison with the values reported in literature for other P450 enzymes.

Protein	β-sheet	3_10_-helix + random	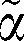 helix	β-turn
rArom	26±1	16±5	37±3	22±3
rArom + substrate	25±4	18±2	38±4	19±2
rArom + inhibitor	22±4	17±2	38±5	23±3
CYP101^a^	22	12	34	31
CYP111^a^	18	20	46	17
CYP102^a^	24	17	38	21
CYP2B4^a^	19	20	35	26
CYP11A1^a^	26	11	36	27

[49].^a^ from ref.

**Figure 3:**
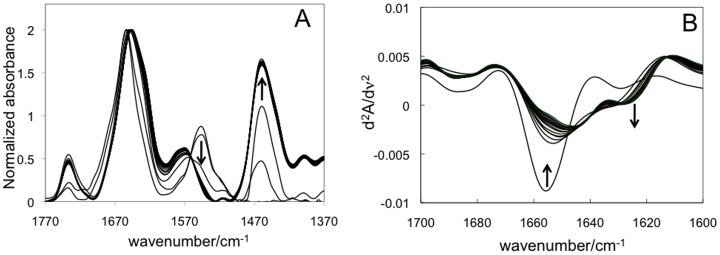
FTIR spectra of rArom during H/D exchange. A) Spectra recorded as a function of time after connecting the sample cell with a ^2^H_2_O-saturated nitrogen flow. The spectra are normalized for the amide I signal. B) Second derivative spectra obtained from the FTIR spectra recorded as a function of time. The signals at 1655, 1623 and 1640 cm^−1^ are assigned to α-helices, β-sheets and random coil, respectively [49].

FTIR spectroscopy was also used to determine the kinetics of H/D exchange at 24°C over a time of 200 minutes (Figure 3). In order to exclude possible denaturation effects, the amide I signal was deconvoluted at the end of each repeat experiment to verify the presence of the same secondary structure elements reported in Table 1. Furthermore, the absorption spectrum in the visible region was checked to verify the presence of the added ligands and the correct folding of the protein. Moreover CO-binding to the reduced protein was performed to confirm the presence of the structurally unmodified P450 enzyme at the end of each experiment.

Figure 3A shows the spectra recorded during H/D exchange for a sample of rArom. Both the amide I and amide II bands are found to shift towards shorter wavenumbers, from 1655 to 1649 and from 1549 to 1455 cm^−1^, respectively.

In order to follow the H/D exchange of the secondary structure elements, the second derivative spectra were generated and the intensity of the signals at fixed wavelength corresponding to the different elements of the secondary structure are followed as a function of time (Figure 3B) [50]. In particular, in line with the assignments found in literature [49] the signal at 1655 cm^−1^ was followed for the α-helices, at 1623 cm^−1^ for the β-sheets and at 1640 cm^−1^ for random coils. The signal of the α-helices is found to decrease and shift toward lower wavenumbers during H/D exchange, while the signal of the β-sheets resulted to increase. The intensity change of each spectrum recorded during H/D exchange for 200 minutes for the 1655, 1623 and 1640 cm^−1^ signals is than plotted as a function of time. Experimental data were found to follow exponential kinetics, as expected for protein deuteration, and were found to better fit to the sum of two exponential decay functions (Figures S2-S4) suggesting the presence of two populations of protons with different exchange rates in all the samples. The values found for the rate constants are in keeping with the literature data [51]–[54] and indicate that the H/D exchange rate constants of the fastest exchanging protons on the protein surface fall in a timescale not detected by the experiment. On the other hand the first one (*k_1_*, Table 1) is assigned to the exchange of flexible partially exposed protons and the second one (*k_2_*, Table 2) to the protons in flexible buried regions [51]–[54]. Statistical analysis of the values shows that significant differences in both *k_1_* and *k_2_* values for α-helices were found in rArom complexed with androstenedione and anastrozole when compared to the free form. Significant differences were observed in both H/D exchange rates (*k_1_* and *k_2_*) of the β-sheets only for the protein complexed with anastrozole.

**Table 2:** H/D exchange rates of the secondary structure elements.

	α-helix		β-sheet		Random coil	
	*k_1_ (min^−1^)*	*k_2_ (min^−1^)*	*k_1_ (min^−1^)*	*k_2_ (min^−1^)*	*k_1_ (min^−1^)*	*k_2_ (min^−1^)*
rArom	0.13±0.01	0.07±0.01	0.22±0.06	0.02±0.001	0.38±0.04	0.02±0.001
rArom+sub	0.09±0.02**	0.02±0.001**	0.21±0.03	0.02±0.001	0.47±0.05	0.02±0.001
rArom+inhib	0.07±0.01**	0.02±0.002**	0.12±0.02**	0.02±0.001*	0.43±0.06	0.01±0.002

**P<0.001 in comparison to the value for rArom.

*P<0.005 in comparison to the value for rArom.

### Fluorescence spectroscopy

**Figure 4:**
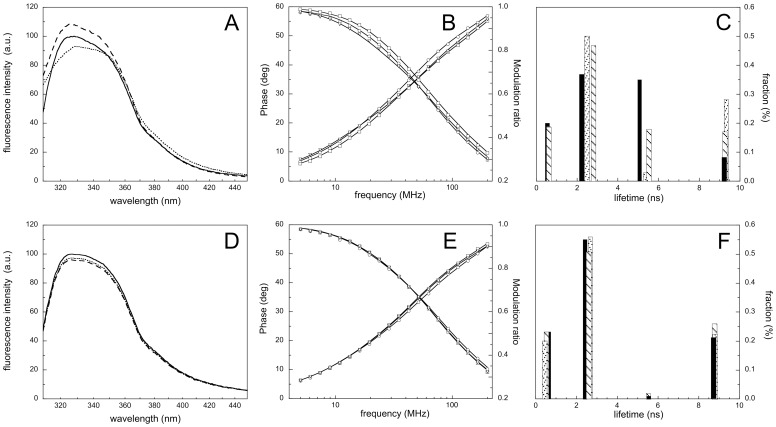
Fluorescence properties of rArom and the mutant Trp224Phe. A) Steady-state fluorescence emission spectra (λ_exc_ = 293 nm) of rArom in the absence (solid line) and in the presence of the substrate androstenedione (dashed line) or the inhibitor anastrozole (dotted line). B) Phase-shift and demodulation data of rArom in the absence (squares), and in the presence of the substrate androstenedione (circles) or the inhibitor anastrozole (triangles). C) Fluorescence lifetimes obtained from the fitting of the experimental data to a four discrete components model for rArom in the absence (black bars), and in the presence of the substrate androstenedione (dashed bars) or the inhibitor anastrozole (dotted bars). Panels D), E) and F) use the same symbols to show the results obtained under the same conditions for mutant Trp224Phe.

The steady-state emission fluorescence spectra of rArom are reported in figure 4. The spectral shape and intensity of the wild-type rArom are strongly influenced by the presence of the substrate or the inhibitor (Figure 4A). Analysis of the protein 3D structure shows that human aromatase contains 5 Trp residues (Trp67, Trp88, Trp141, Trp224 and Trp239), but only one (Trp224) is part of the active site. In particular, it is located in helix F and it entertains van der Waals contacts with the substrate androstenedione [14]. For this reason the mutant Trp224Phe was generated and, in this case, no significant changes were detected upon ligands binding (Figure 4D). When compared to the wild-type rArom, this mutant shows a higher dissociation constant for both androstenedione (4.6±0.3 µM) and anastrozole (0.59±0.03 µM) and therefore 50 µM of androstenedione and 3 µM of anastrozole were added to reach saturation. The activity of the mutant resulted also decreased by 35% compared to the non-mutated protein.

Taking advantage of the sensibility of time resolved fluorescence techniques, we further characterized the effects observed in the steady-state measurements, performing a set of parallel experiments using the phase-shift and demodulation methodology (Figure 4B and 4E).

A complex, multi-exponential emission decay is necessary to adequately fit the data. In particular, according to the lower chi-square value (Table S1), the best fit was obtained using a four components model, with lifetimes (τ) centered at 0.55 (τ_1_), 2.2 (τ_2_), 5.1 (τ_3_), and 9.2 (τ_4_) ns for the ligand-free enzyme (Figure 4C).

The addition of the 10 µM androstenedione or 1 µM anastrozole to rArom results in a shift of τ_2_ and τ_3_ towards longer values (Figure 4C) and a significant decrease of the fraction percentage of the emitting species of the third component.

Due to its presence in the ligand-binding site and its ability to closely interact with the bound ligand, it is reasonable to expect that Trp224 is contributing the most to the τ_2_ and τ_3_ components that are significantly affected by ligand binding.

When a four discrete components model is used to fit the data of the Trp224Phe mutant, the fraction of τ_3_ results to be decreased to a negligible signal of less than 1%, demonstrating that this component is specific for Trp224. The addition of the androstenedione or anastrozole to the Trp224Phe mutant does not significantly perturb the three remaining τ components (Figure 4D). These data prove that the change of τ_3_ observed upon ligand addition to rArom is related to the presence of Trp224. As changes in fluorescence lifetimes are known to be linked to changes in the dynamics of the fluorophores that generates them, these results indicate that the dynamic of Trp224 is affected by ligand binding. The presence of the τ_2_ component in the Trp224Phe mutant indicates that this component is due to more than one Trp residue. It is, in fact, well established that a single Trp residue can contribute to more components of τ [55]. It is important to note that Trp224 is located in helix F, that in other cytochromes P450 is found to be flexible to allow substrate entrance [56]. From these observations it follows that also in rArom the F-helix is involved in some conformational changes that are key to allow access of the ligand into the binding site. In alternative, it could be argued that the substitution of the tryptophan residue with a phenylalanine produces a more compact local tertiary structure, making the other tryptophans insensitive to the presence of both substrate and inhibitor. This possible explanation is in keeping with the different K_D_ values found for wild-type rArom and the Trp224Phe mutant.

## Discussion

**Figure 5:**
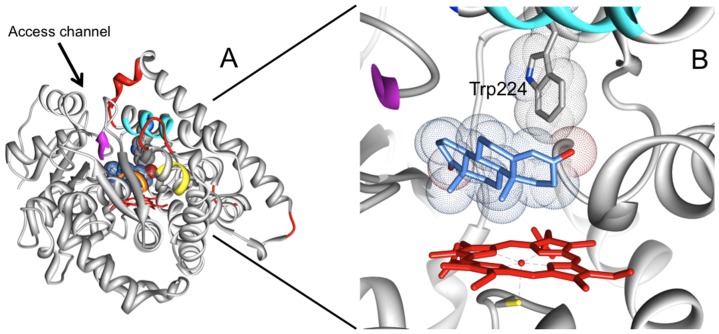
Access channel of rArom for the substrate androstenedione. A) Overall structure of rArom (PDB ID 4KQ8). The most flexible regions according to the MD simulations [18] are shown in red. The parts of the secondary structure elements forming the access channel are shown in yellow (helix E), cyan (helix F), orange (helix)I, magenta (β-strand 9). B) Zoom in the active site: the heme is shown in red, the Van der Waals surface of androstenedione (blue) and Trp224 (grey) are also shown.

Ever since the publication of the crystal structure of human placental aromatase that revealed a very compact nature of the enzyme in the presence of the substrate androstenedione, one of the key questions is how the protein undergoes conformational changes to allow the entrance of different ligands in the tight active site [57]. The difficulty of obtaining a purified protein without the addition of any ligand hindered experiments aiming at addressing this question. For this reason one of the key aspects of this present work is the successful achievement of a recombinant system that allows to purify human aromatase in the absence of any substrate/inhibitor. Ligand-free thermal unfolding data of recombinant aromatase reveal T_m_ values similar to those reported before for other mammalian cytochromes P450 [45]. Furthermore, the addition of the substrate androstenedione results to increase the T_m_ by 3°C suggesting the presence of protein-substrate interactions that stabilise the protein. This is consistent with the crystal structure showing the presence of both hydrophobic and polar interactions between androstenedione and different residues of the catalytic cleft of the protein. However, this kind of stabilization is not observed in the presence of the inhibitor anastrozole that is bound to the protein through direct coordination of the heme iron by the nitrogen atom of the triazole moiety [34]. The protein complexed with anastrozole also displays a different behaviour in the H/D exchange experiments with respect to the form complexed with the substrate androstenedione. The H/D exchange rates for -sheets are lower in the presence of the inhibitor, suggesting a perturbation of these elements. Furthermore, time resolved fluorescence data show that the fluorescence dynamics of Trp224, located in helix F (Figure 5B), is less affected by anastrozole binding, in comparison to the binding of androstenedione. In fact, in the case of the substrate, the componet τ_3_ almost completely disappears whereas, in the presence of the inhibitor anastrozole, is only reduced by 50%. Analysis of the crystal structure reveals that Helix F (cyan in Figure 5) is part of the substrate access channel that, according to molecular dynamics simulations, encounter hinge-bending motions likely to be responsible for its intrinsic flexibility [18]. The channel is also formed by helix I, part of helix E and β-sheet 3 (Figure 5A).

Unfortunately to date there is no crystal structure of the enzyme in complex with the inhibitor, preventing a direct comparison of the different effects that inhibitor or substrate binding have on the crystal structure. However MD simulations [18] show that the substrate access channel is formed by part of helix F, I and E and β-sheet 3 (Figure 5A). This confirms that both elements of secondary structure (α-helices and β-sheets) are involved, as found by ATR FT-IR findings, and that helix F is involved in anastrozole binding as indicated by fluorescence spectroscopy.

In conclusion, this work reports the first study of the dynamics and flexibility of ligand-free aromatase. Taken together our results show that aromatase flexibility is decreased by the addition of a ligand: different H/D exchange rates for β-sheets and localized dynamics effect on Trp224 suggest a different effect on the conformation given by the substrate androstenedione and the inhibitor anastrozole. The different degree of flexibility observed between the ligand-free and -bound forms is compatible with the hypothesis of the presence of an open conformation in the ligand-free form.

## Supporting Information

Figure S1 **Far-UV circular dichroism spectra of a sample of rArom at increasing temperatures.**
(TIF)

Figure S2 **Deuteration of α-helices as function of time.** A) Data for ligand-free rArom and fitting to a double exponential decay (first exponential decay R^2^ = 0.9983, second exponential decay R^2^ = 0.9912). B) rArom in the presence of androstenedione with fitting to a double exponential decay (solid line, R^2^ = 0.9989) and a monophasic exponential decay (dashed line, R^2^ = 0.9840). C) rArom in the presence of anastrozole with fitting to a double exponential decay (first exponential decay R^2^ = 0.9994, second exponential decay R^2^ = 0.9960).
(TIF)

Figure S3 **Deuteration of β-sheets as function of time.** A) Data for ligand-free rArom and fitting to a double exponential decay (first exponential decay R^2^ = 0.9989, second exponential decayR^2^ = 0.9964). B) rArom in the presence of androstenedione with fitting to a biphasic exponential decay (solid line, (first exponential decay R^2^ = 0.9998, second exponential decay R^2^ = 0.9952). C) rArom in the presence of anastrozole with fitting to a biphasic exponential decay (first exponential decay R^2^ = 0.9980, second exponential decay R^2^ = 0.9864).
(TIF)

Figure S4 **Deuteration of random coil as function of time.** A) Data for ligand-free rArom and fitting to a double exponential decay (R^2^ = 0.9957) and a monophasic exponential decay (dashed line, R^2^ = 0.8608). B) rArom in the presence of androstenedione with fitting to a biphasic exponential decay (solid line, R^2^ = 0.9976) and a monophasic exponential decay (dashed line, R^2^ = 0.8403). C) rArom in the presence of anastrozole with fitting to a biphasic exponential decay (R^2^ = 0.9865) and a monophasic exponential decay (dashed line, R^2^ = 0.8322).
(TIF)

Table S1 Chi-square values of the fitting of the time resolved fluorescence data to different exponential components.
(DOCX)
