# Supplementary figures and images for: Dynamics and Flexibility of Human Aromatase Probed by FTIR and Time Resolved Fluorescence Spectroscopy
Source: PLoS One. 2013 Dec 11;8(12):e82118. doi: 10.1371/journal.pone.0082118 (PMC3859599; doi:10.1371/journal.pone.0082118)

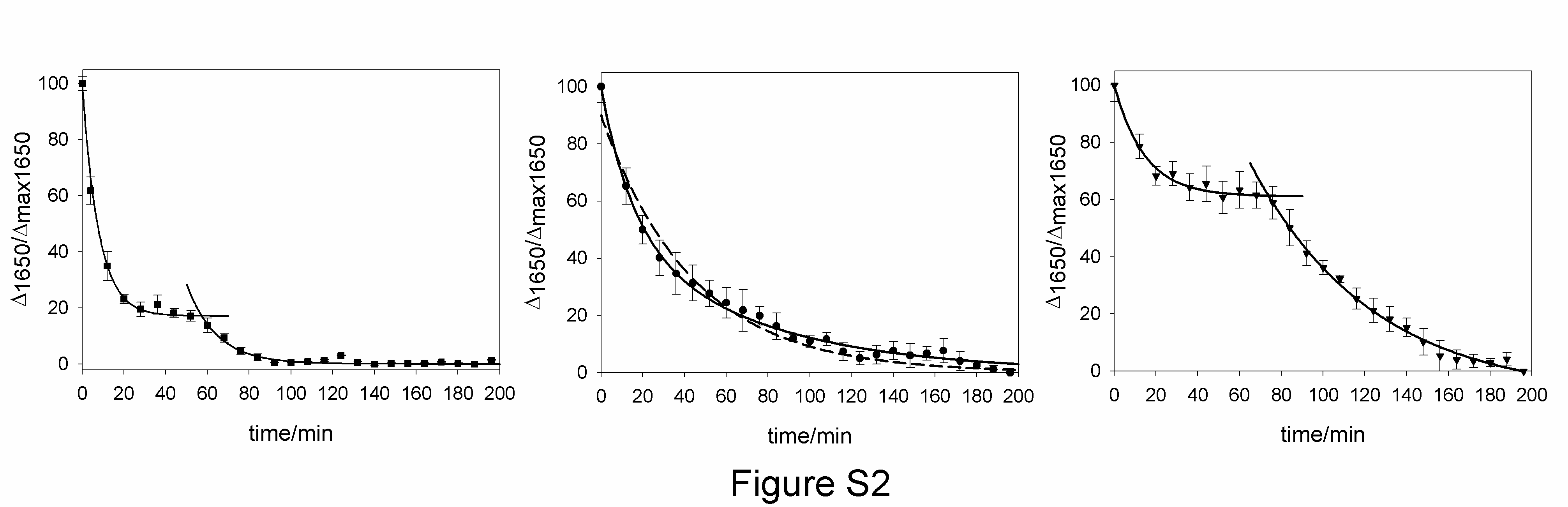

Supplement: Figure S2 — Deuteration of α-helices as function of time. A) Data for ligand-free rArom and fitting to a double exponential decay (first exponential decay R2 = 0.9983, second exponential decay R2 = 0.9912). B) rArom in the presence of androstenedione with fitting to a double exponential decay (solid line, R2 = 0.9989) and a monophasic exponential decay (dashed line, R2 = 0.9840). C) rArom in the presence of anastrozole with fitting to a double exponential decay (first exponential decay R2 = 0.9994, second exponential decay R2 = 0.9960). (TIF) [file pone.0082118.s002.tif]

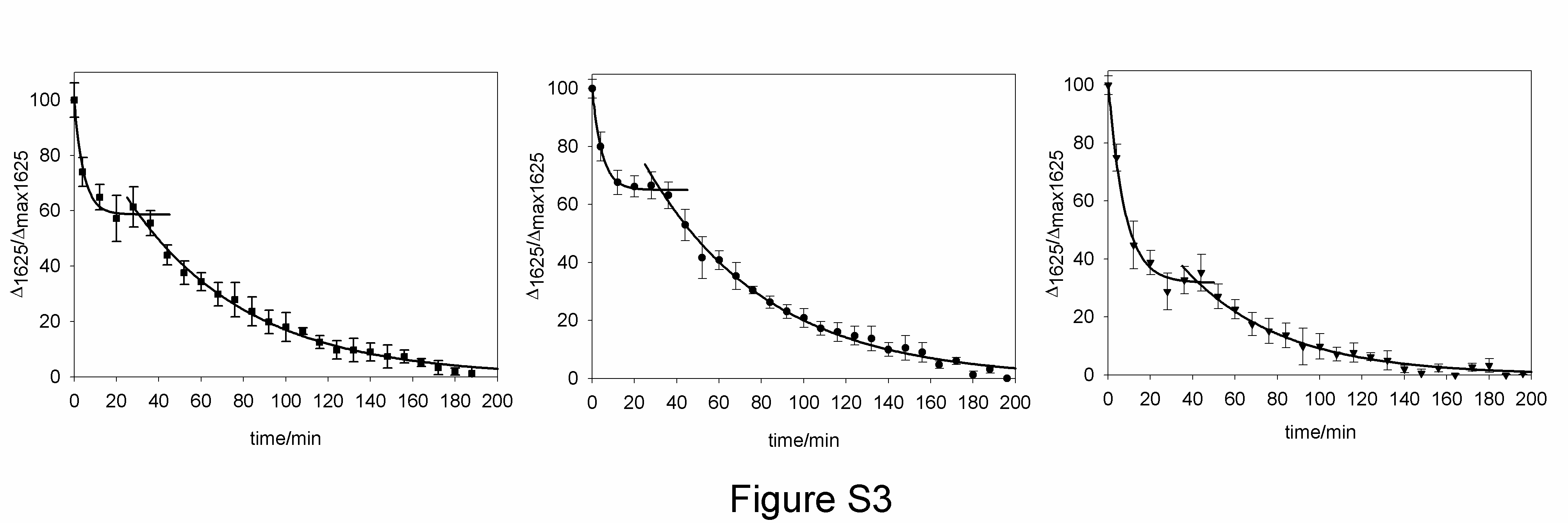

Supplement: Figure S3 — Deuteration of β-sheets as function of time. A) Data for ligand-free rArom and fitting to a double exponential decay (first exponential decay R2 = 0.9989, second exponential decayR2 = 0.9964). B) rArom in the presence of androstenedione with fitting to a biphasic exponential decay (solid line, (first exponential decay R2 = 0.9998, second exponential decay R2 = 0.9952). C) rArom in the presence of anastrozole with fitting to a biphasic exponential decay (first exponential decay R2 = 0.9980, second exponential decay R2 = 0.9864). (TIF) [file pone.0082118.s003.tif]

Table S1.


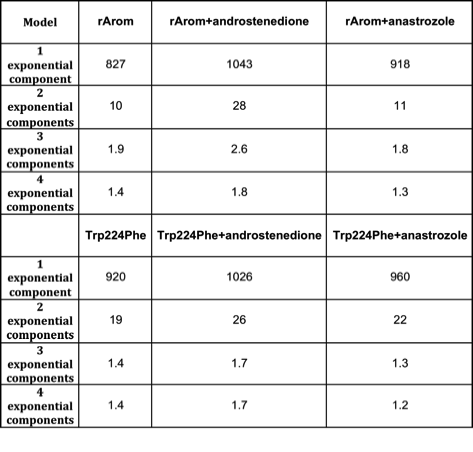

Supplement: Table S1 — Chi-square values of the fitting of the time resolved fluorescence data to different exponential components. (DOCX) [file pone.0082118.s005.docx]
